# Nowcasting unemployment rate during the COVID-19 pandemic using Twitter data: The case of South Africa

**DOI:** 10.3389/fpubh.2022.952363

**Published:** 2022-12-02

**Authors:** Zahra Movahedi Nia, Ali Asgary, Nicola Bragazzi, Bruce Mellado, James Orbinski, Jianhong Wu, Jude Kong

**Affiliations:** ^1^Africa-Canada Artificial Intelligence and Data Innovation Consortium (ACADIC), Laboratory for Industrial and Applied Mathematics, York University, Toronto, ON, Canada; ^2^Africa-Canada Artificial Intelligence and Data Innovation Consortium (ACADIC), The Advanced Disaster, Emergency and Rapid Response Program, York University, Toronto, ON, Canada; ^3^Africa-Canada Artificial Intelligence and Data Innovation Consortium (ACADIC), Schools of Physics, Institute for Collider Particle Physics, University of the Witwatersrand, Johannesburg, South Africa; ^4^Africa-Canada Artificial Intelligence and Data Innovation Consortium (ACADIC), The Dahdaleh Institute for Global Health Research, York University, Toronto, ON, Canada

**Keywords:** sentiment analysis, social media, Twitter data, Google Mobility Index, unemployment rate, labor market, COVID-19, South Africa

## Abstract

The global economy has been hard hit by the COVID-19 pandemic. Many countries are experiencing a severe and destructive recession. A significant number of firms and businesses have gone bankrupt or been scaled down, and many individuals have lost their jobs. The main goal of this study is to support policy- and decision-makers with additional and real-time information about the labor market flow using Twitter data. We leverage the data to trace and nowcast the unemployment rate of South Africa during the COVID-19 pandemic. First, we create a dataset of unemployment-related tweets using certain keywords. Principal Component Regression (PCR) is then applied to nowcast the unemployment rate using the gathered tweets and their sentiment scores. Numerical results indicate that the volume of the tweets has a positive correlation, and the sentiments of the tweets have a negative correlation with the unemployment rate during and before the COVID-19 pandemic. Moreover, the now-casted unemployment rate using PCR has an outstanding evaluation result with a low Root Mean Square Error (RMSE), Mean Absolute Percentage Error (MAPE), Symmetric MAPE (SMAPE) of 0.921, 0.018, 0.018, respectively and a high R^2^-score of 0.929.

## Introduction

The novel coronavirus known as “severe acute respiratory syndrome-related Coronavirus type 2” (SARS-CoV-2), responsible for the “Coronavirus Disease 2019” (COVID-19) pandemic, was first detected in the metropolitan city of Wuhan, Hubei Province, mainland China, in late December 2019. Since then, it quickly spread around the globe, causing more than 481 million infections and 6 million deaths, as of March 30, 2022 (1, 2). The World Health Organization (WHO) officially declared the COVID-19 outbreak, initially, as a Public Health Emergency of International Concern (PHEIC) on January 30, 2020, and, later as a global pandemic on March 11, 2020 (3).

Since then, countries have enforced non-pharmaceutical interventions (NPIs) to curb the diffusion of the virus and prevent its spread, including lockdowns and different levels of restrictions. Even though effective both from a clinical and epidemiological perspective, consecutive rounds of NPIs have had devastating effects on the economy and caused bankruptcy to many companies and businesses (4). As a result, many people and individuals have lost their jobs and countries are experiencing economic recession (5). To better manage the economic impacts of the pandemic on the economy and people, it is highly important to have complete, reliable, and real-time information about the effects of the pandemic on the unemployment rate as one of the key macroeconomic indicators (6, 7).

Traditional census methods that are used by most countries to generate unemployment data are often conducted on a seasonal or annual basis (8, 9). While this provides sufficient information for public policies in normal situations, these methods lack the details and urgency that are required for decision-making during a disaster, such as a pandemic. Census data often use questionnaires on a sample of households to collect employment data. Despite using new technologies in data collection (such as online surveys) and analysis, censuses are still expensive, time- and resource-consuming, and difficult to handle. The census method faces many other challenges and limitations such as privacy concerns, low public cooperation, errors caused by response burden, cybersecurity attacks (e.g., denial of service), and missing out on hard-to-reach populations. Migration, homelessness, and nomadism may result in under- or over-registration, making collected data not representative of the entire population. Low levels of literacy and language issues may cause some people to struggle with the census forms and fail to provide correct information.

Due to such difficulties, the unemployment rate in South Africa is also estimated quarterly. In contrast, social media data is readily available. Statistics and demographic information can be easily extracted and processed in real-time. Many of the problems and limitations of the classical census approach do not exist when data are extracted and estimated using social media (10, 11). Twitter data has the potential to present socio-demographics, statistics, and textual information/content that can be exploited to estimate/model macroeconomic indicators, like the unemployment rate (12). Moreover, approximately 82% of the Twitter users in South Africa are of working ages (16–54 years). About half of them are women (56%) and half of them are men (44%) (13). Finally, retrieving data from Twitter is not expensive and time-consuming, and it does not require manpower and administrative personnel. With several lines of code, data can be quickly accessed: with the streaming and full archive search endpoints, data is available in real-time, and in several days at maximum, respectively.

As unemployment increases, it becomes a common concern, and everyone generally talks about it more. On the other hand, as unemployment decreases, everyone is less bothered by it, and it is less talked about on social media. As a result, the aggregated data derived from social media reflects the unemployment situation and can potentially be used to estimate the statistics (14–19). Moreover, applying sentiment analysis which is a way of classifying text for extracting qualitative insights gives additional information that could be further used for machine learning-based prediction (20, 21).

Access to socio-economic data such as unemployment rates is very critical for rapid and effective decision-making and public health policies, during devastating disasters such as the still ongoing COVID-19 pandemic. In the present study, we propose a method for understanding and estimating unemployment rates during COVID-19 using social media, particularly Twitter data (22). As previously mentioned, accessing data extracted from social media is fast, easy, and low-cost. It can be done in real-time and does not have the difficulties and limitations of census-based methods.

Social media provide a large amount of data about users and their interactions about a given subject, thereby, offering researchers new opportunities for research (23–26). Twitter as a pervasive social media is widely used for understanding economic behavior and measuring its metrics (27, 28). It is also one of the most popular social media in South Africa (29, 30). With the implementation of NPIs, such as lockdowns and the closure of workplaces and public areas, people spend even more of their time on social media (31).

In this paper, we aim to examine how Twitter data can be used to collect qualitative and quantitative information about the unemployment rate and how unemployment is lived and experienced in South Africa as a case study. This could be beneficial to policymakers, especially during disasters such as the COVID-19 pandemic as it can capture and report rapid changes in unemployment in real-time rather than seasonally or annually. This work may enable policymakers to understand the current situation of the labor market and react in terms of policies. Accordingly, the main contribution of this study includes:

- Using the quantity of the tweets to understand how people experience unemployment.
- Using the quality of the tweets (or sentiments) to understand how people feel about unemployment.
- Nowcasting and finding the missing data on the unemployment rate using the quantity and quality of the tweets.

## Background and literature review

Social media, especially Twitter, has long been used for investigating economic issues. Authors in (32) searched for tweets with hashtags for different keywords on jobs and gathered tweets sent by popular users in the United States. Sentiment analysis showed that most of the tweets had negative sentiments. In (33) a sentiment-based model was designed with 0.6787 accuracies for tweets, news articles and movie reviews and concluded that the sentiment scores were correlated with economic indexes such as the exchange rate. Although social media has long been used for studying economic issues and related concerns, very few studies have considered using social media to understand the unemployment rate. One of the first works that used Twitter to estimate the unemployment rate is presented in (14). In this paper, 19.3 billion tweets were gathered from July 2011 to November 2013 on unemployment in the United States. Principal Component Analysis (PCA) was used to reduce the dimension of the dataset. The unemployment rate of the United States was then estimated using the principal components. A similar approach was proposed in (15) for studying the correlation between the number of unemployment-related tweets and the unemployment rate in Greece. Sentiment analysis has not been considered in these two studies to improve the results further. Ryo in (17) analyzed the sentiments of Korean tweets, blogs, and news articles, and used sentiments to predict the unemployment rate with autoregression analysis (like ARIMAX and ARX). The Twitter dataset was found to have the lowest error. The authors in (18) used Twitter data to study the unemployment and employment rates in the United States. Using sentiment analysis, they found out that negative and positive sentiments peak when people lost or gain jobs. They also used sentiment analysis to predict the unemployment rate of the United States. Authors in (19) built a linear model to predict employment and unemployment rates using tweets from the United States. Although the papers mentioned above have presented novel methods for studying the unemployment rate using social media, they have not investigated unemployment rate changes during a disaster such as the COVID-19 pandemic.

Authors in (16) hydrated a Twitter dataset and used it to study the correlation between the number of unemployment-related tweets and the unemployment rate and track the unemployment rate of the USA during the COVID-19 pandemic. However, because of the limitations in their dataset, they were not able to properly understand how the unemployment rate changed over time.

Some social media-related studies have focused on the labor market flow during the COVID-19 Pandemic. Authors in (34) used Twitter to study the effect of different factors on reopening sentiments. They found that people with low income, low education level, high housing rent, and in the labor force are more positive about reopening. In (35) Twitter was used to study the economy of the United States during the COVID-19 pandemic. In this work, the Area Deprivation Index (ADI) of different geographical locations was used to assess the economic situation of people. It concluded that in low resource areas, people were more concerned with economic hardship while in high resource areas people were more focused on public health. In (36) data from Twitter and newspaper articles were used to study economic uncertainty in the United Kingdom and the United States during the COVID-19 pandemic. Numerical results show that with the COVID-19 pandemic, a huge uncertainty jump was found in economic-related indicators such as business growth, GDP growth, and stock market volatility.

These papers have investigated the effect of the COVID-19 pandemic on the economy. However, they do not consider studying and estimating the unemployment rate using social media. The main contribution of this study is to fill the existing gaps in using social media data to understand, analyze, and estimate the unemployment rate during the pandemic using a combination of methods. This combination has significantly improved the classical method for estimating the unemployment rate.

## Materials and methods

Our complete code can be found at (37).

**Figure 1:**
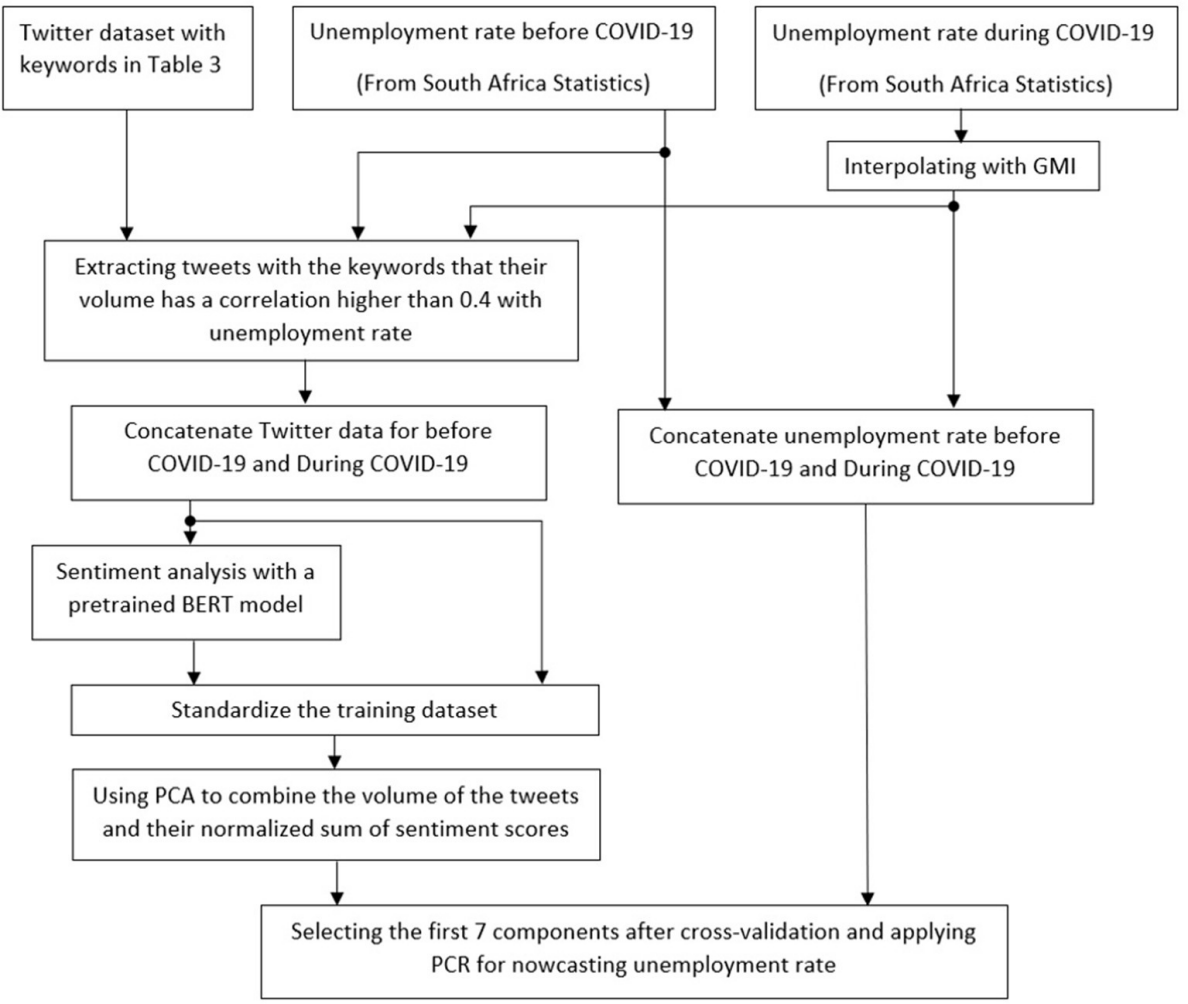
The overall architecture of the research.

The unemployment rate for South Africa is estimated in four steps. In the first step, relative keywords are selected to collect Twitter data. In the second step, missing unemployment data is estimated using Google Mobility Index (GMI). In the third step, sentiment analysis is performed to achieve further information about the labor market conditions. Finally, in the fourth step Principal Component Regression (PCR) is used to estimate the unemployment rate from the number of unemployment-related tweets. The overall architecture of the project is presented in Figure 1.

We evaluate our method using four different metrics, Root Mean Square Error (RMSE), Mean Absolute Percentage Error (MAPE), Symmetric Mean Absolute Percentage Error (SMAPE), and coefficient of determination (R^2^-score), which are presented in equations 1–4.


(1) $$\begin{array}{lll} RMSE & = & \sqrt{\frac{\sum_{i=1}^{n}{\left(A_{i}-P_{i}\right)}^{2}}{n}} \end{array}$$

(2) $$\begin{array}{lll} MAPE & = & \frac{1}{n}\overset{n}{\underset{i=1}{\sum }}\left|\frac{A_{i}-P_{i}}{A_{i}}\right| \end{array}$$

(3) $$\begin{array}{lll} SMAPE & = & \frac{1}{n}\sum_{i=1}^{n}\frac{\left|A_{i}-P_{i}\right|}{\frac{\left|A_{i}|+|P_{i}\right|}{2}} \end{array}$$

(4) $$R^{2}=1-\frac{\sum_{i=1}^{n}{\left(A_{i}-P_{i}\right)}^{2}}{{\sum^{\text{​}}}_{i=1}^{n}{\left(A_{i}-\underline{A}\right)}^{2}}$$

Where *n* is the number of tested values, A is the actual unemployment rate, is the mean unemployment rate, and *P* is the predicted values.

### Data collection

All the geotagged tweets posted from South Africa, except for retweets, until Nov 30th, 2021, for certain keywords are retrieved using full archive search of the Twitter Academic Researcher account. Tweets are cleaned, i.e. mentions (@username), URLs, and punctuations are removed. Records that include only URLs become null/nan after cleaning and are deleted. Our method requires a dataset of tweets from real and genuine accounts (38). Therefore, we aim to remove as many tweets posted by bots and fake accounts as possible. Since most tweets that are created by bots include URLs, many of them are deleted after removing null/nan records (39). To further take out tweets created by bots and fake accounts we examine the number of followers and followings of the authors. Generally, users that have a very large or small number of followers to followings ratio are broadcasters or spammers, respectively. Genuine users have a followers to followings ratio close to one (40). Therefore, by removing tweets that their authors have a followers to followings ratio greater than *t1* = 10 or smaller than *t2* = 0.1, more tweets from fake accounts are excluded. It is worth mentioning that decreasing or increasing the thresholds t1 or t2, respectively, degraded the performance of the regression model.

It is worth mentioning that minors are not excluded from the dataset. Since minors can also post how they or their friends and family, e.g., parents, guardians, etc., are experiencing the unemployment, their comments and sentiments could add useful information to the model and increase the accuracy of the PCR. Next, the Term Frequency (TF) of the keywords are found over time using Equation 5.


(5) $$\begin{array}{l} TF=\frac{\left|tweet_{k}\right|}{\left|tweet_{total}\right|} \end{array}$$

Where tweet_k_ is the number of tweets that include keyword k, and tweet_total_ is the total number of tweets. Using the TF of the keywords, the Pearson correlation of each keyword over time with the unemployment rate is calculated. In the economy, correlations higher than 0.4 and 0.7 are considered moderate and strong, respectively (41). To avoid overfitting our estimation model, we chose the keywords which have a correlation higher than 0.4, before and during the COVID-19 pandemic for training the nowcasting model.

We build our final dataset using the selected keywords that have a correlation higher than 0.4 with the unemployment rate before and during the COVID-19 pandemic. The cleaned tweets are suitable and used for performing Natural Language Processing (NLP) such as sentiment analysis. The dataset is divided into two parts. The first part contains tweets up to March 31st, 2020, and the second part contains tweets from April 1st, 2020 up to Nov 30th, 2021. The first part is used to analyze the tweets and their sentiments before the COVID-19 pandemic and the second part is used for the COVID-19 pandemic period.

To make sure the volume of the tweets truly correlates with the unemployment rate in the long run, we go further into history and gather the tweets as early as possible. Geotagged tweets with our keywords are available from June 2009. However, due to the low volume (lower than ten tweets per month) of tweets between June 2009 and June 2010, we leave out the tweets from this period. The number of tweets have a moderate to high correlation with the quarterly and interpolated unemployment rate of South Africa from July 2010 to Nov 2021, respectively. Moreover, we compared the number of tweets for each province with the unemployment rate of that province since July 2010 and find a moderate to high correlation for all of them. The results are presented in Appendix A, supplementary files. Previous works have used the change in geolocation of the geotagged tweets in a certain time period to identify mobility and travel (42, 43). We find all the geolocations of the tweets sent by each 144,809 users in 1 year to recognize travelers/non-residents. Similar to (43), we used the place field of the json file that is returned by the Twitter API to discover the province of the user when sending the tweet. Users which have posted from multiple provinces are identified as travelers. The most frequent geo-province associated to a user is considered as the primary location of that user, and where the user is residing, or at least working. Thus, the most frequent geo-province associated to each user is assigned to all the tweets sent by that user in that specific year. If more than one province has the greatest number of occurrences, the self-reported location of the user is taken as the primary location of the user (43). The primary province of a few users (2 users on average) that are not identifiable in each year, are taken out from the dataset for that particular year, when nowcasting the unemployment rate of the provinces. By this method the correlation between the volume and sentiment of the tweets and unemployment rate, as well as the estimation using PCR in different provinces increased (see Appendix A, supplementary files).

### Data preprocessing

**Figure 2:**
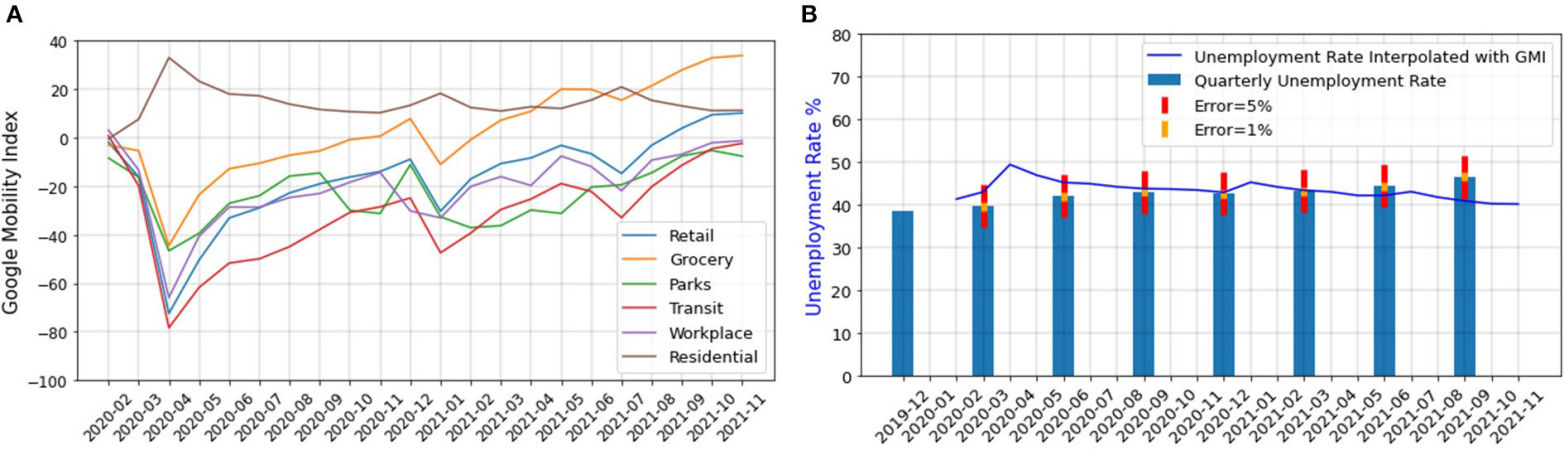
**(A)** GMI for different categories of places over time for South Africa (28), **(B)** Unemployment rate of South Africa interpolated with GMI.

The real unemployment data for South Africa is provided on a seasonal basis (44), and it is calculated in two different ways. In the first method, an individual is considered unemployed during an interview if (1) the individual was not employed in the seven days before the interview, (2) the individual is ready to work within a week of the interview, and (3) has actively taken some steps to look for a job or start a self-employed business, 4 weeks before the interview. In the second method, the third condition is relaxed (45). Since people do not normally look for new jobs or start a business during a lockdown (even if they are jobless), in our work, we use the second definition. This expanded definition of unemployment aligns with the definition in many other countries (45). However, due to on and off rounds of lockdowns the unemployment rate has changed rapidly during COVID-19, and the quarterly unemployment rate is not capable of capturing the rapid fluctuations. We thus use GMI to interpolate the census unemployment rate during the COVID-19 pandemic (46–48). According to the International Labor Organization (ILO), the unemployment rate can be approximated using GMI (47). GMI shows the movement trends over time and space in six different categories of places namely, retail and recreation, grocery and pharmacies, parks, transit stations, workplaces, and residential. It was released to the public on Feb 15th, 2020 and will be removed after the pandemic (46). Since GMI is only temporarily available, it cannot be used to estimate the unemployment rate after the pandemic. However, Twitter data is always available and can be used for understanding, nowcasting, and even interpolating the unemployment rate. Figure 2A shows the indexes of GMI categories over time for South Africa.

Because a residential activity is not a work-related function and its index has a negative correlation with the rest of the indexes, we exclude it from our analysis. We average the indexes of all other categories and used linear regression to interpolate the unemployment rate of South Africa from GMI data. Equation 6 contains the results obtained from fitting a linear regression model to the GMI data.


(6) $$unemp=-0.1354\times \underline{GMI}+41.1134$$

*GMI* represents the GMI averaged over all the categories except for the residential places and *unemp* is the interpolated unemployment rate. Figure 2B shows the quarterly unemployment rate (26) interpolated using the GMI for South Africa. In this figure, the error bars show that the highest error occurs in the estimation of the unemployment rate for August 2021.

To evaluate the goodness of the fit, we used the SMAPE Metric. This metric which is shown in Equation 3 is a value between 0 and 2, with 0 indicating a perfect fit and 2 showing the worst fit possible. Generally, a SMAPE value lower than 0.1 shows a really good regression fit (49). We found a value of 0.05196 for SMAPE which indicates that our simple linear regression model captures the GMI data quite well.

### Data labeling and sentiment analysis

Sentiment analysis is an NLP procedure that classifies text based on its affective states. Sentiment analysis is done using a pretrained Bidirectional Encoder Representations from Transformers (BERT) model (50, 51). The model is trained using a large Twitter dataset (52, 53). We randomly choose 200 tweets from our dataset and manually label them as negative, neutral, or positive. We find that the model has 0.69 accuracy on our dataset. Based on how negative, neutral, or positive a tweet is, the machine assigns a score between−1 and 1 to the tweet. Negative, neutral, and positive tweets have a score close to−1, 0, and 1, respectively (54).

Unemployment has increased during the COVID-19 pandemic, and everybody even the young population that are not in the work force have suffered from it (55, 56). Even in wealthier families, children and adolescents that are not in the working-age have experienced anxiety and depression due to financial and economic crises (57). Their experience may not be as negative and acute as adults, but the economic recession caused by lockdowns and unemployment is reflected in their comments and sentiments, as well (55). In addition, in poorer families, economic crises may cause adolescents to quit school and enter the labor market to supplement their household economy (58). These negative sides and impacts of the pandemic and lockdowns are reflected in the tweets, and we capture them by performing sentiment analysis. The result efficiently increases the performance of the model for nowcasting the unemployment rate.

The normalized sum of the sentiment scores over time is calculated for the two parts of the dataset and compared with unemployment rate, before and during the COVID-19 pandemic. Moreover, the sentiment classes and scores for different provinces are calculated and compared. The two datasets are concatenated to train the PCR model and estimate the unemployment rate. From the 1182,632 different tweets, 289,738 tweets belong to the second part of the dataset (COVID-19 pandemic period), and the rest belong to the first part (pre-COVID-19 pandemic period). Figure A.1 in Appendix A in supplementary files shows the word-cloud generated for our dataset.

### Model development and validation

**Figure 3:**
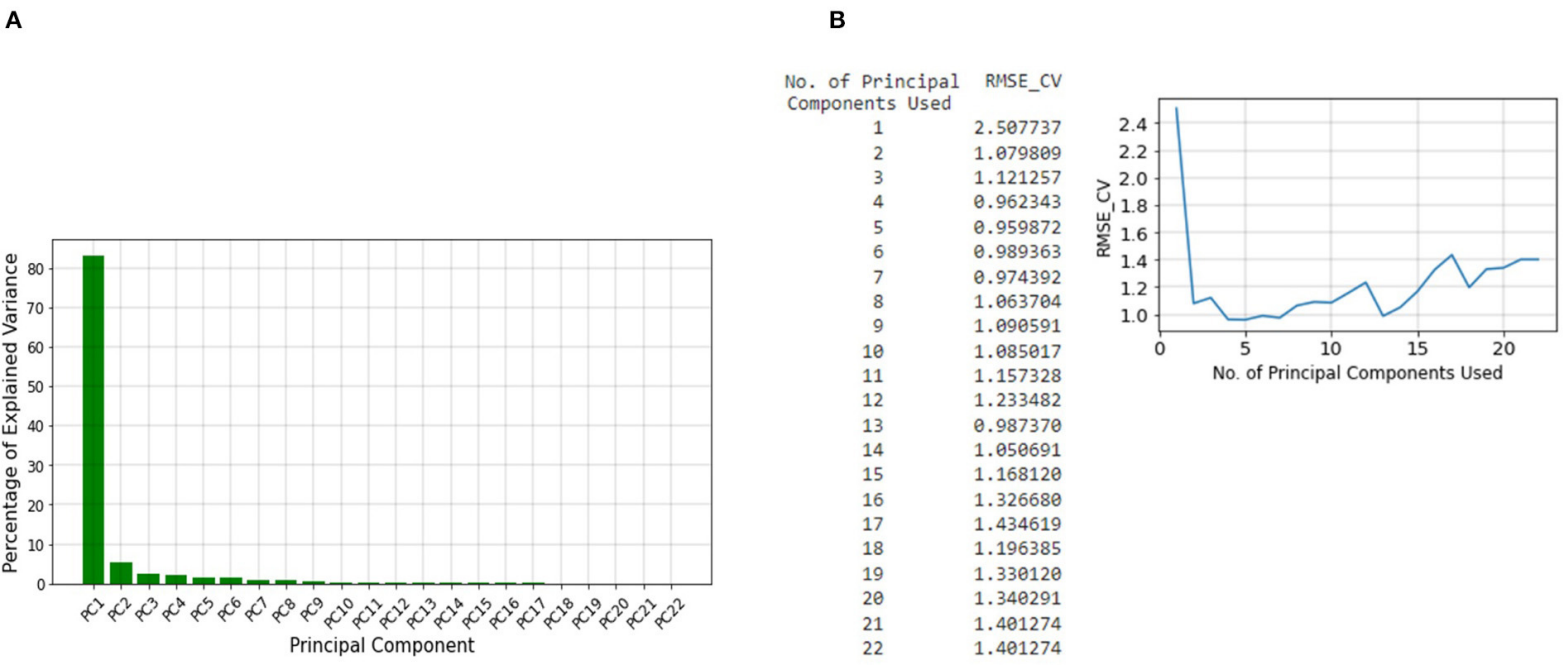
**(A)** The percentage of explained variances for the different principal components, **(B)** Cross-validation RMSE for linear regression with different number of components.

After concatenating our datasets, the number of tweets over time for the whole dataset and different keywords are found and stored in a vector. Next, since the normalized sum of the sentiment scores over time have a negative correlation with the unemployment rate, it is inverted and stored in a separate vector. These vectors which make up the training set, are standardized to improve the performance of the regression model. The unemployment rate is also stored in a different vector and used as labels for the PCR. The PCR method is essentially a linear regression model on the principal components of the training dataset (59). Therefore, PCA is applied to all of the vectors of the training dataset, and twenty-two different principal components are found. According to Figure 3A, the first component accounts for more than 80% of the variance. However, according to Figure 3B, the cross-validation Root Mean Square Error (RMSE) indicates that the least error is obtained when five of the principal components are used for linear regression. Therefore, we use linear regression with the first five principal components in our model.

## Results

The method is implemented using Python 3 in Google Colaboratory (60). Using the vectorization feature of python, we are able to process our large dataset in no time. However, the sentiment analysis part which requires Graphics Processing Unit (GPU) takes more than 3 hours to execute (37).

### Quantity of the tweets

Table A.1 in Appendix A in the supplementary files shows the keywords used for retrieving tweets (14), their correlations with the unemployment rate, their *p*-values, and whether they are selected for tracing the unemployment rate and training the PCR model.

**Figure 4:**
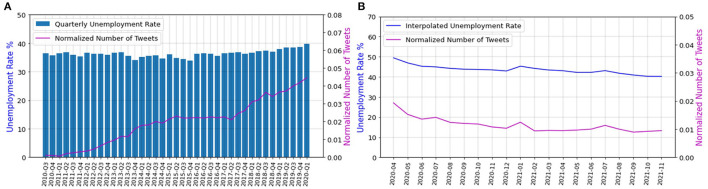
**(A)** Correlation between the unemployment rate and the number of employment-related tweets before the COVID-19 pandemic, **(B)** Correlation between the unemployment rate and the number of employment-related tweets during the COVID-19 pandemic.

The total dataset is 53% and 90% correlated with the unemployment rate and has a *p*-value of 4 × 10^−4^ and 3.76 × 10^−8^ before and during COVID-19 pandemic, respectively. Figures 4A,B show the correlation between the number of tweets in the total dataset and the unemployment rate before and during the COVID-19 pandemic, respectively.

According to these results, the employment-related tweets gathered using our selected keywords are significantly correlated with the unemployment rate of South Africa, during and before COVID-19. Next, the two datasets for before and during COVID-19 pandemic are concatenated. Figure A.2A in Appendix A, supplementary files, shows that the number of tweets in the concatenated dataset is also highly correlated with the unemployment rate. Thus, Twitter data may be used to estimate the unemployment rate in real time.

### Sentiment classification

**Figure 5:**
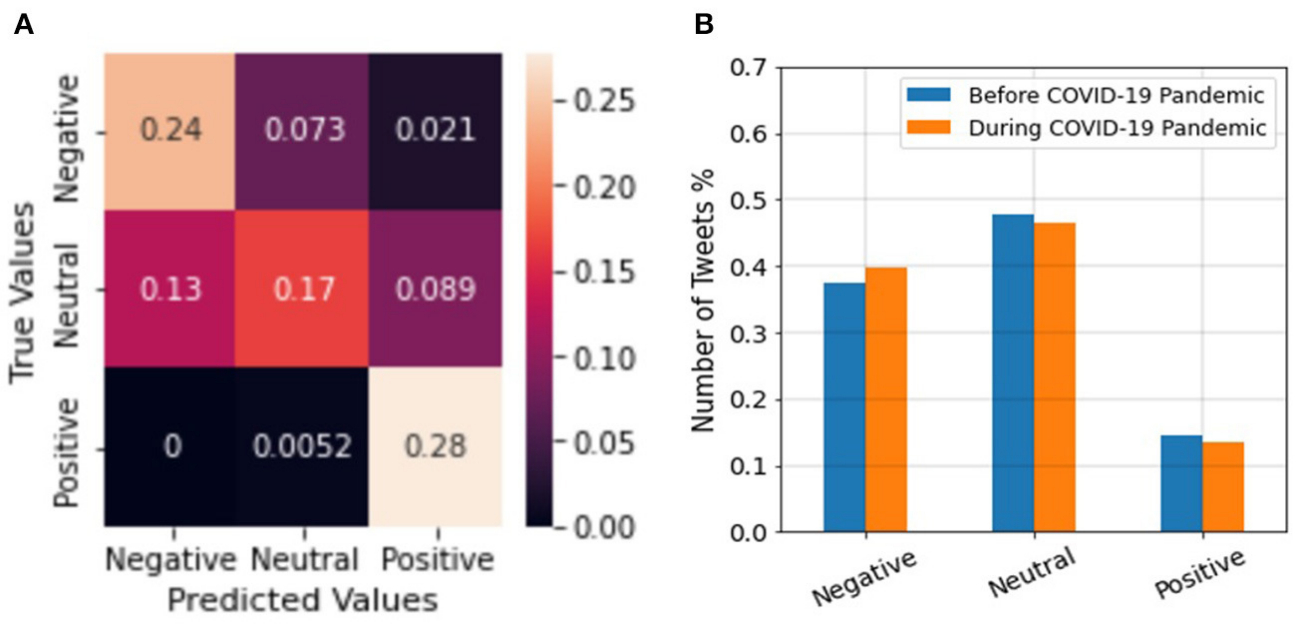
**(A)** Confusion matrix of the sentiment analysis model, **(B)** Percentage of tweets from different sentiment classes.

Figure 5A shows the confusion matrix of the pretrained model tested on our labeled dataset. The diameter of the confusion matrix indicates that the accuracy of the model is 69%. Table 1 shows the precision, recall, and f1-score of the model. The average of the parameters on different polarities also suggests that the accuracy of the model on our dataset is approximately 69%. Moreover, Figure 5A and Table 1 also show that tweets from negative and positive polarities are better recognized than tweets with neutral polarity. The reason could be that tweets with neutral sentiment may carry a mixture of positive and negative polarity and therefore are more difficult to distinguish.

**Table 1:** Evaluation metrics of the pretrained model tested on our labeled dataset.

**Sentiment polarity**	**Precision**	**Recall**	**F1-score**
Positive	0.72	0.98	0.83
Neutral	0.68	0.44	0.53
Negative	0.66	0.72	0.69

Figure 5B shows the number of negative, neutral, and positive employment-related tweets before and during the COVID-19 pandemic. As shown in Figure 5B, there are more tweets with negative sentiments than with positive sentiments. This is as expected since the dataset is on unemployment-related tweets. Moreover, it can be seen in Figure 5B that sentiment classes are more negative and less positive during the COVID-19 pandemic compared to before. This is in-line with previous research on social media sentiments during COVID-19 (61). As the COVID-19 pandemic started, people found more free time during the lockdowns to spend in social media. In addition, they were able to communicate with friends and family while social distancing (62). However, the microblogging sentiments were not always more positive than before COVID-19 pandemic (61, 63). Authors in (62, 63) found that the most dominant emotion of tweets regarding topics related to COVID-19 were fear, anticipation, and trust. This means that they were scared of the pandemic circumstances, yet hopeful that new solutions will be unearthed for prevention and recovery. Moreover, emotions regarding economy have been very negative during the COVID-19 pandemic (64, 65). The study in (64, 65) show that at the beginning of the pandemic investors became very fearful and uncertain of the stock market trends and trading. The sentiments regarding oil price were dominantly fear at the beginning of the pandemic as well (65). Finally, it is stated in (65) that Twitter sentiments around job and employment continued to be more optimistic in March 2020. People were hopeful that everything will go back to normal after the lockdowns. However, since April 2020, emotions have been increasingly becoming less optimistic and more anxious and annoyed regarding the labor market.

**Figure 6:**
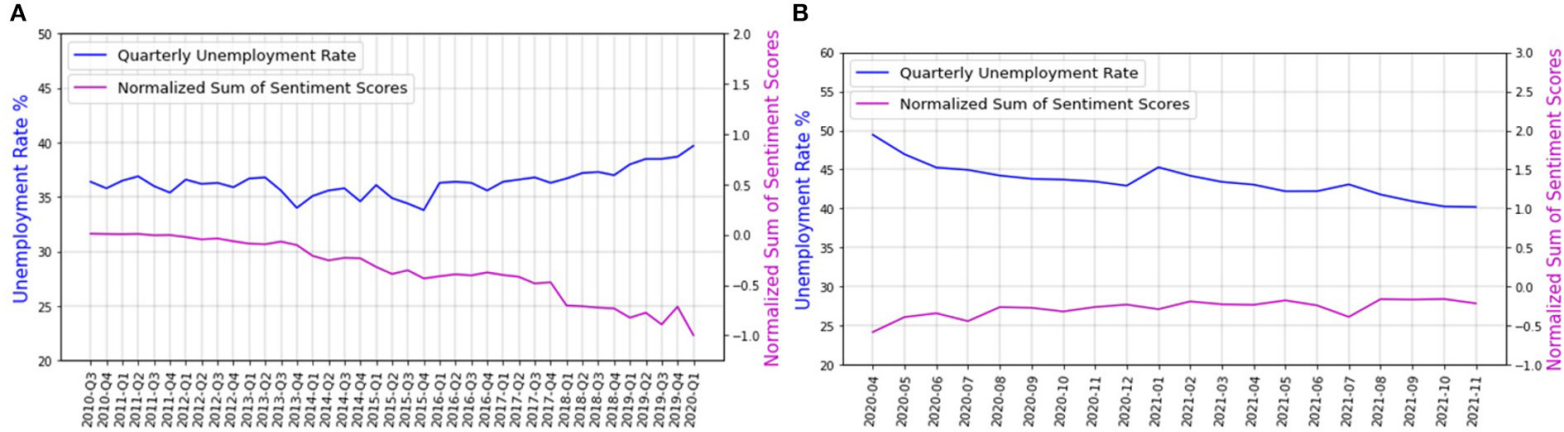
The distribution of the sum of sentiment scores over time **(A)** before and **(B)** during the COVID-19 pandemic.

We compare the normalized sum of sentiment scores with the unemployment rate, during and before COVID-19. Figure 6 shows the distribution of the normalized sum of the sentiment scores (A) before and (B) during the COVID-19 pandemic, over time. Table 2 shows the correlation and the *p*-value of sentiment scores with the unemployment rate for the first and second part of the dataset, i.e., before and during the COVID-19 pandemic, and the correlation between the concatenated dataset and the unemployment rate.

**Table 2:** The correlation between sentiment scores and the unemployment rate as well as that between the concatenated dataset and the unemployment rate.

	**Normalized sum of sentiment scores**		**Concatenated dataset**
	**Unemployment rate before COVID-19 pandemic**	**Unemployment rate during COVID-19 pandemic**	**Unemployment rate**
Correlation	−0.6	−0.86	0.72
*P*-value	< 0.001	< 0.001	< 0.001

According to Figure 6 and Table 2, the sentiment scores have a high negative correlation with the unemployment rate, during and before the COVID-19 pandemic. This could be interpreted to mean that the higher the unemployment rate, the more negative the sentiments of the employment-related tweets. The sentiments can be used to qualitatively analyze employment-related tweets, to understand how dissatisfied people are with unemployment.

### Nowcasting the unemployment rate

**Figure 7:**
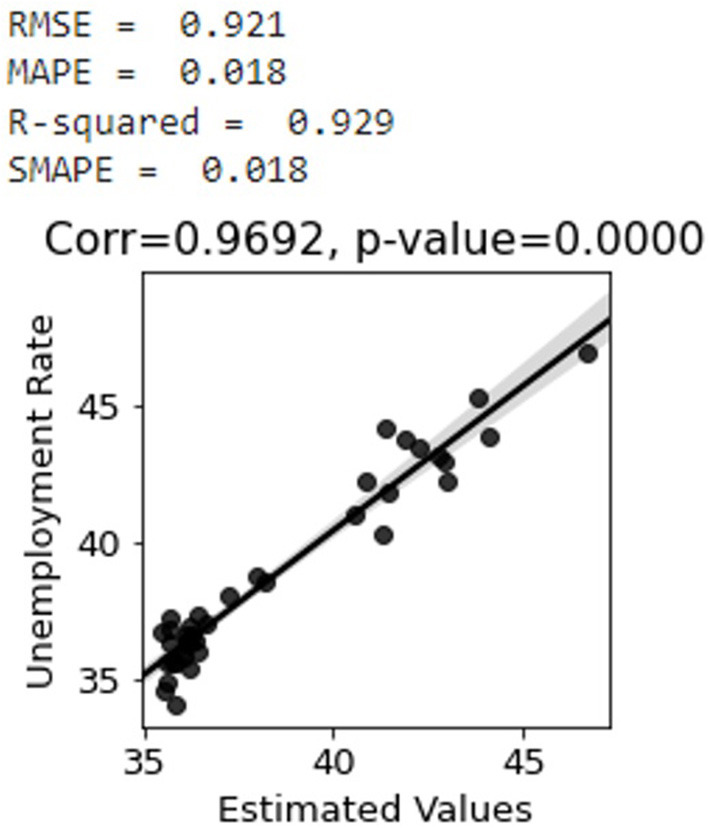
Correlation between unemployment rate and estimated values of the whole country with 95 percentiles.

The sentiment scores are next inverted to have a positive correlation with the unemployment rate. Two-thirds of the dataset is used for training the PCR model and the remaining portion is used for testing. According to Figure 7, the predicted values of the unemployment rate are very well–correlated with the actual values. The SMAPE, RMSE, MAPE, and coefficients of determination R^2^ metrics in Figure 7 are calculated using Eq. 1-4 (31). As shown in Figure 7, the trained model has R^2^-score of 0.93 and SMAPE of 0.01 which is very outstanding.

**Figure 8:**
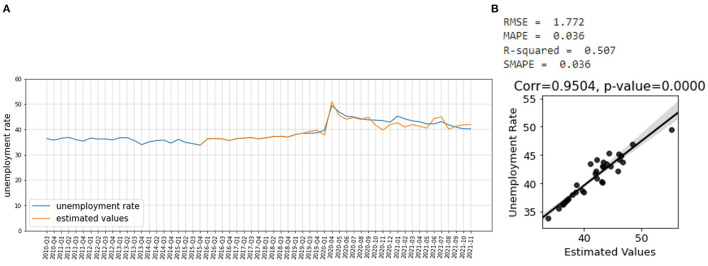
**(A)** Estimated unemployment rate, **(B)** Correlation between the estimated values of the unemployment rate during COVID-19 and the actual unemployment rate for the whole country with 95 percentiles.

Figure 8A shows that the estimated unemployment rate matches the actual unemployment rate. Figure 8B shows that the estimated unemployment rate is well–correlated with the actual unemployment rate, during the COVID-19 pandemic. The model has an R^2^-score of 0.51 and SMAPE of 0.03 which shows that it has a good effect size and performs very well (49).

**Figure 9:**
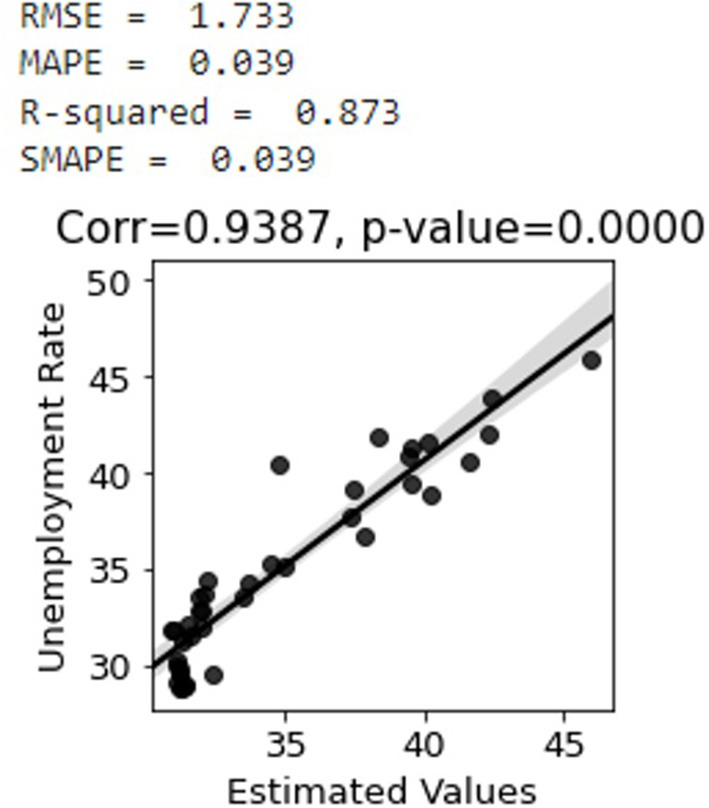
Correlation between unemployment rate and estimated values of Gauteng province with 95 percentiles.

We have also used PCR to nowcast the unemployment rate of different provinces. Figure 9 shows the correlation between the actual unemployment rate of Gauteng and the estimated values when about two-third of the data is used for training the PCR model and one-third is used for prediction.

According to Figure 9, the model for Gauteng has a SMAPE of 0.03 and an R^2^-score of 0.89 which indicates a pretty good prediction.

**Figure 10:**
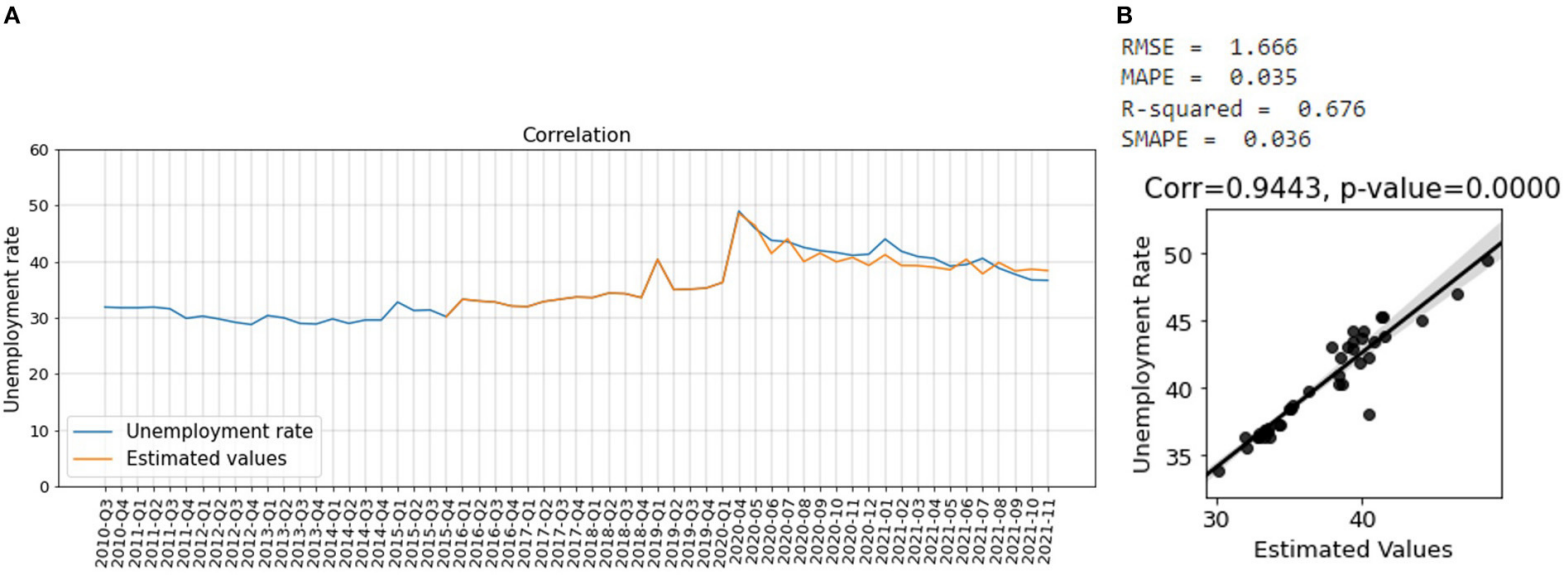
**(A)** Estimated values of unemployment rate of Gauteng during COVID-19 pandemic matches the actual unemployment rate, **(B)** Correlation between the estimated values of the unemployment rate during COVID-19 and the actual unemployment rate for Gauteng province with 95 percentiles.

Moreover, we use one-third of the data before the COVID-19 pandemic to train the PCR model and then use the trained model to nowcast the unemployment rate during the COVID-19 pandemic for different provinces in South Africa. Figure 10A shows that the predicted values closely follow the actual unemployment rate for Gauteng. Figure 10B shows the correlation between the actual and predicted values of the unemployment rate for Gauteng. We obtain a SMAPE value of 0.03 and R^2^-score of 0.68, which indicates a good prediction.

The results for the rest of the provinces can be found in Appendix A in supplementary files.

## Discussion

In this paper, we use social media to nowcast the unemployment rate of South Africa. We find that the number of tweets on certain keywords has a high correlation with the unemployment rate in South Africa. Moreover, the social sentiments of the tweets are negatively correlated with the unemployment rate. Social media provide a large amount of data about users and their interactions about a given subject, thereby, offering an unconventional data source for data-driven policy decisions. It is turning into the primary place where people share their thoughts and daily activities. In addition to what people express on social media, an investigation of their underlying attitudes can help inform policies. Some of these conversations on social media are employment-related.

In this study, we show that certain keywords extracted from employment-related tweets can be used to nowcast the unemployment rate. The selected keywords correlate with the unemployment rate for all the years considered. Therefore, it is very likely that the number of tweets gathered with these keywords will keep on correlating with the unemployment rate, in the future. Moreover, the fact that the normalized sum of the sentiment scores of the tweets gathered with these keywords has a strong negative correlation with the unemployment rate verifies that these keywords can reflect the unemployment rate. As the unemployment rate increases, people begin to talk about it on the social media, in a negative way, and the selected keywords can pick this reflection.

Our PCR method for estimating the unemployment rate using the number of tweets on the selected keywords and the normalized sum of their sentiments has an SMAPE and R^2^-score of 0.01 and 0.93, respectively.

**Figure 11:**
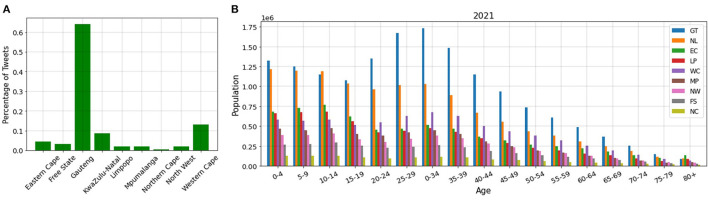
**(A)** Number of tweets in each province, **(B)** Distribution of age population on different provinces.

In conclusion, our PCR method can estimate the unemployment rate of a country very well. This is very valuable as it allows us to remove the barriers and difficulties of the census methods and estimate the unemployment rate in real time. Furthermore, to make sure that the dataset gathered truly captures the unemployment rate and can be used to nowcast it in the long run, we find the number of tweets belonging to each province in South Africa and stratify the provinces based on age and industry. Figure 11A shows the number of tweets of each province. Most of the tweets come from urban provinces, namely, Gauteng, KwaZulu-Natal, and Western Cape (66). These provinces contain more than 85% of the tweets. Other provinces which are considered rural account for < 15% of the tweets.

However, when we study the distribution of age and industry population in different provinces, we find that (1) most of the people of working ages (20–60 years old) live in the urban provinces (Gauteng, KwaZulu-Natal, and Western Cape), and (2) the most populated industries are in the urban provinces. Figure 11B shows the distribution of the age population in different provinces for 2021. We depict this diagram for 2020, 2019, and 2018 and find that they have a similar distribution (67). The diagrams can be found in our complete code (37). As can be seen in this figure, the population of working ages (20–60 years old) for KwaZulu-Natal and Gauteng is almost 2 times and 3 times more than the rural provinces, respectively. Moreover, after these two provinces, working ages are more populated in Western Cape, compared to the rural provinces.

In South Africa specifically, about 82% of Twitter users are of the working ages (16–54 years) (8, 13, 68). Based on the above, we conclude that the tweets that we have gathered are dominantly from people in their working ages, talking about their economic condition and therefore, the volume of the tweets does represent the unemployment situation of the country.

**Figure 12:**
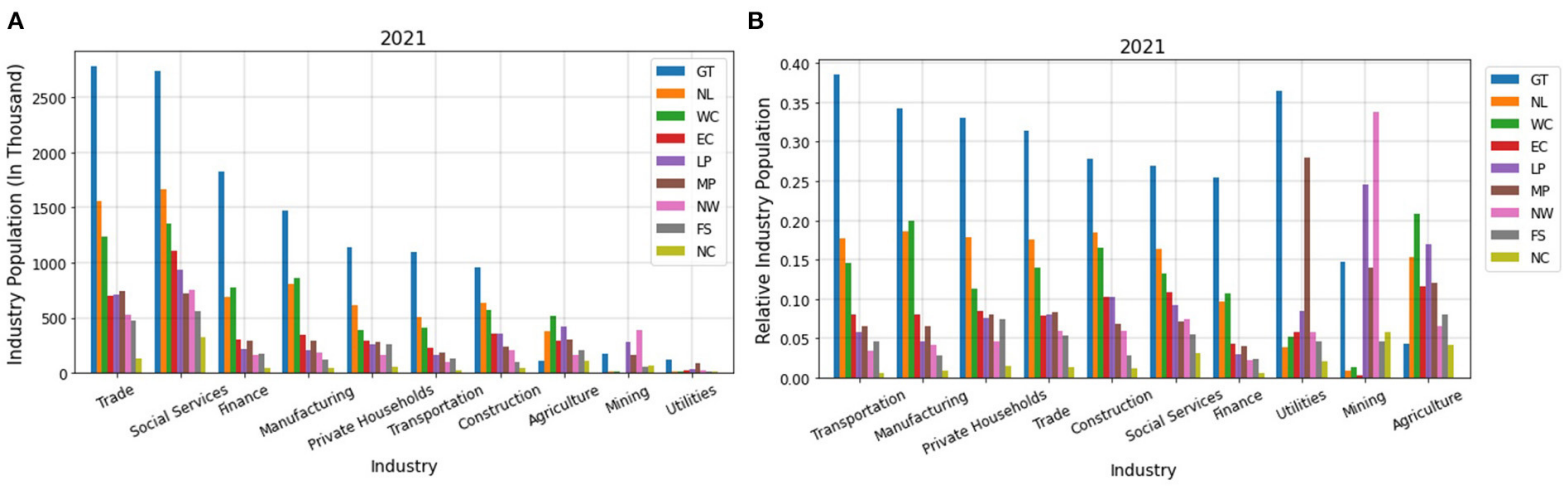
**(A)** Distribution of industry population on different provinces, **(B)** Population of industries in different provinces divided by the total population of that industry.

Next, we find the distribution of industry population in different provinces. Figure 12A shows the distribution of industry population in different provinces and Figure 12B shows the population of a given industry divided by the whole population of that industry for different provinces, for 2021. These diagrams for 2020, 2019, and 2018 are very similar to 2021 and can be found in our complete code (37).

As can be seen in Figure 12B, in all the industries, except for utilities, mining, and agriculture, most of the population live in Gauteng, KwaZulu-Natal, and Western Cape. The population living in Gauteng for these industries is almost 2 times, and even 3 times in some cases, more than that of the other provinces. Among the rural provinces, most of the population for these industries live in Eastern Cape which has the highest number of tweets according to Figure 12B. For utilities, mining, and agriculture, the population in rural provinces is considerable, however, according to Figure 12A the population working in these industries is very small. Therefore, we conclude that the number of tweets is highly attributed to the population working in different industries and reflects the economic situation of different sectors.

In conclusion, what we are capturing by tweet volume is associated with the unemployment rate of the country and considering that it has always correlated with the unemployment rate of the country since the beginning of Twitter, it will most probably represent the unemployment rate of the country in the long run. Results in Appendix A in supplementary files show that the number of Tweets in each province has a moderate to strong correlation with the unemployment of that province which also shows that we have gathered the tweets using the right keywords.

**Figure 13:**
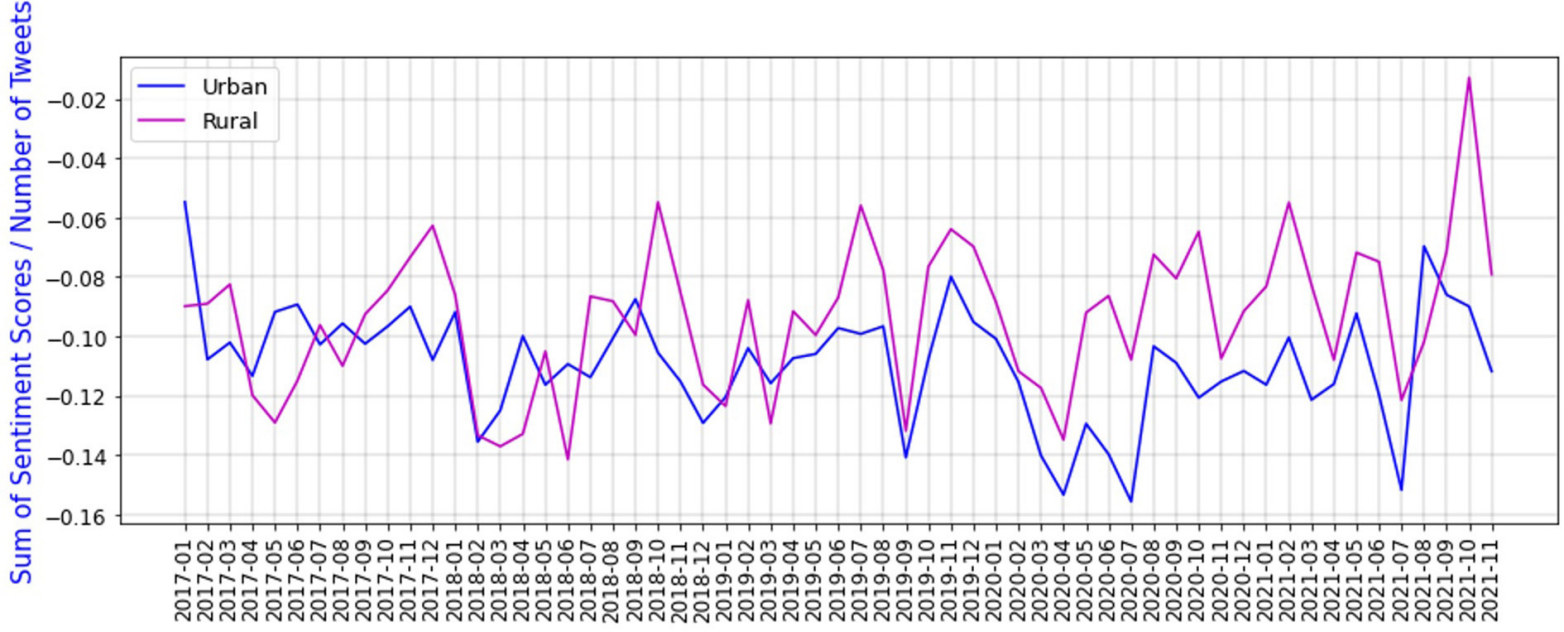
Sum of sentiment scores divided by number of tweets for urban and rural areas.

We also calculate the sum of sentiment scores divided by the number of tweets, over time in urban and rural areas of South Africa. Figure 13 shows the sum of sentiment scores divided by the number of tweets in urban and rural areas, since 2017.

According to Figure 13 sentiments for urban areas are noticeably lower during COVID-19 pandemic compared to before it. One probable reason could be that during the COVID-19 pandemic, the economy was devastated, and most industries and people from working age groups are located in urban areas. Therefore, the sentiments of urban areas are evidently lower than rural provinces. This is another finding that shows we have gathered the right data from Twitter, and most probably our method can be used to nowcast unemployment rate in the long run.

## Limitations

There are many limitations related to Twitter data that prohibit us from training a perfect model. As previously mentioned, generally, only 15% of online adults, which are mostly 18–29 years old and some minorities, regularly use Twitter. Certain populations, urban/suburban residents, affluent householders, and mobile users, and are more likely to use Twitter (69). As a result, a great portion of the public is left out of consideration. Moreover, 95% of Twitter users never geotag. Among those who consider geotagging their tweets, only about 1% allow most of their tweets to be geotagged. Basically, very passive, and very active users who, respectively post <50 and more than 1,000 tweets per year do not allow most of their tweets to be geotagged. Only moderate users who have 50 to 1,000 tweets per year, frequently allow their tweets to be geotagged. Therefore, a vast number of tweets cannot be used (70, 71). Essentially, among geotagged tweets, only those that are in English can be of use. This is especially crucial when studying multilingual countries such as South Africa. Officially, 11 different languages are spoken in South Africa (72). However, we are only able to gather and analyze English tweets for tracing and nowcasting the unemployment rate.

## Conclusion

In this paper, social media, particularly, Twitter is traced to estimate the unemployment rate of South Africa in real-time. Since in South Africa the unemployment rate is measured quarterly, this method can be used to find the missing information on the unemployment rate, as well. Moreover, this method can provide the unemployment rate statistics in real-time, and without the difficulties faced using the traditional approach. Finally, this information can be highly valuable for analyzing labor market flow when facing disasters such as a pandemic.

The normalized sum of sentiment scores over time before and during the COVID-19 pandemic has a strong negative correlation with the unemployment rate. We combine the number of tweets on different keywords, and the sentiment scores and use PCR to nowcast the unemployment rate. The results show that the estimated unemployment rate is well–correlated with the actual unemployment rate.

One contribution to the future work of this project is to use social media to estimate other economic metrics such as inflation rate, job vacancy rate, labor force participation rate, and part-time working rate. Another work that can be done is to use social media to forecast economic metrics such as the unemployment rate. Different methods or techniques of time series prediction or data mining and machine learning algorithms can be used to forecast these metrics. This can be extremely useful for disaster management response and recovery. Finally, since other media, especially images and videos make up a large portion of social media, new methods need to be proposed to process social media content further.

## Data availability statement

The original contributions presented in the study are publicly available. This data can be found here: https://github.com/Jdkong/Nowcasting_Unemployment and the code is available at: https://colab.research.google.com/drive/1O4NidnStzSGmc-RdJLcUB1NTl5viEcRy?usp=sharing.

## Author contributions

JK and ZN designed research and collected data. All authors conducted literature search, analyzed data, and wrote the paper. All authors contributed to the article and approved the submitted version.

## Funding

This research is funded by Canada's International Development Research Centre (IDRC) and Swedish International Development Cooperation Agency (SIDA) (Grant No. 109559-001).

## Conflict of interest

The authors declare that the research was conducted in the absence of any commercial or financial relationships that could be construed as a potential conflict of interest.

## Publisher's note

All claims expressed in this article are solely those of the authors and do not necessarily represent those of their affiliated organizations, or those of the publisher, the editors and the reviewers. Any product that may be evaluated in this article, or claim that may be made by its manufacturer, is not guaranteed or endorsed by the publisher.
