# Supplementary material for: Nowcasting unemployment rate during the COVID-19 pandemic using Twitter data: The case of South Africa
Source: Front Public Health. 2022 Dec 2;10:952363. doi: 10.3389/fpubh.2022.952363 (PMC9757491; doi:10.3389/fpubh.2022.952363)
Supplement: Supplementary file 1 [file Data_Sheet_1.docx]

Supplementary Material

# Appendix A

A Twitter dataset was gathered with the keywords in Table A.1 using Twitter API academic researcher account. It is shown in Table A.1 that the keywords that had a correlation higher than 0.4 with unemployment rate before and during COVID-19 pandemic where selected for the final dataset which included more than 1million tweets. Figure A.1 shows the word-could of our dataset.


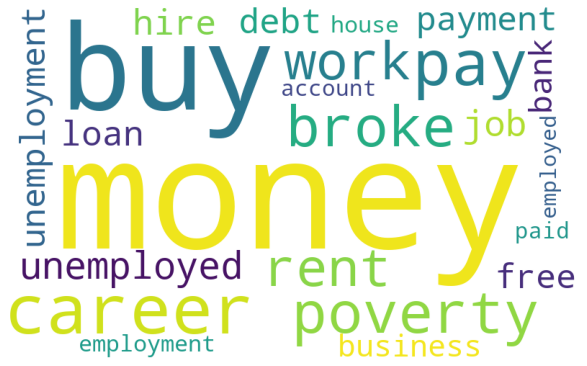


**Figure A.1:** The most frequent words of our dataset

To verify that the number of tweets is truly associated with the unemployment rate, in the long run, we find the correlation between the number of tweets and the unemployment rate from July 2010, until Nov 2021. Moreover, we find the correlation of the tweets in each province with the unemployment rate of that province, in this period. The result is shown in Figure A.2 (A-J) and Table A.2.


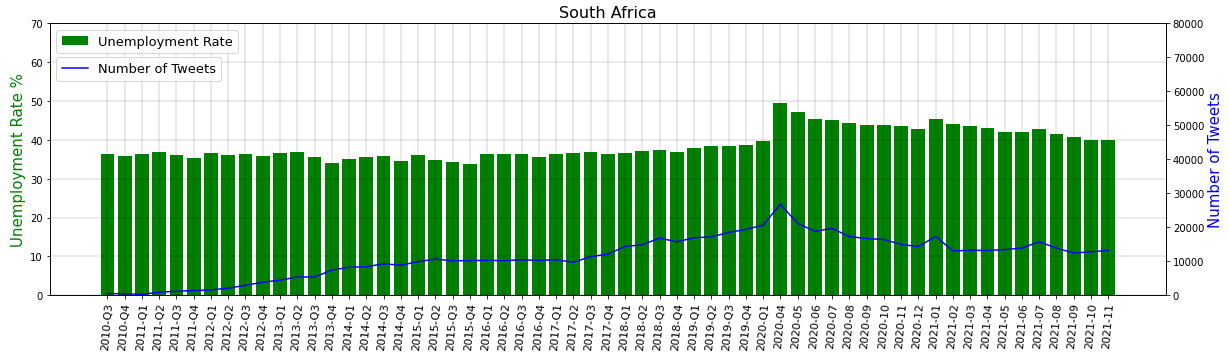


(A) South Africa


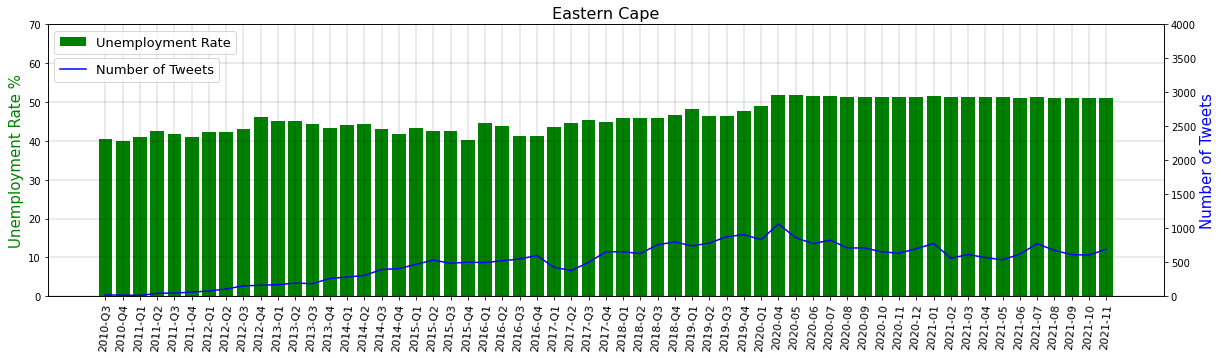


(B) Eastern Cape


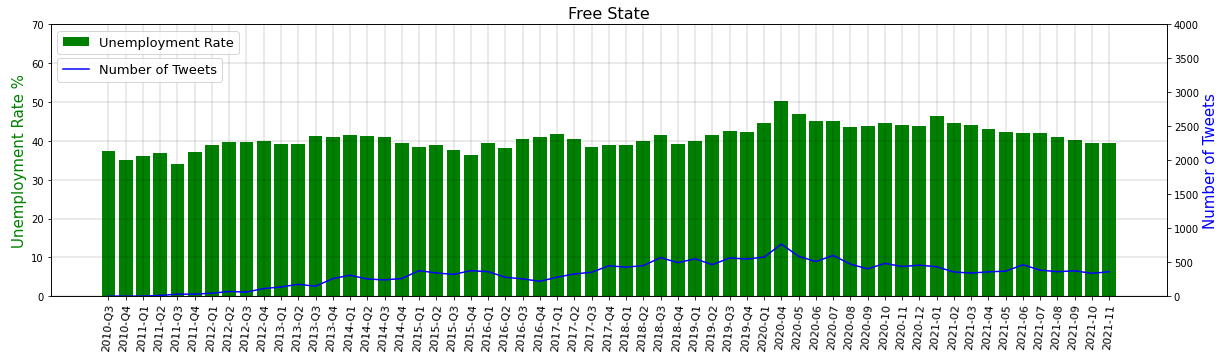


(C) Free State


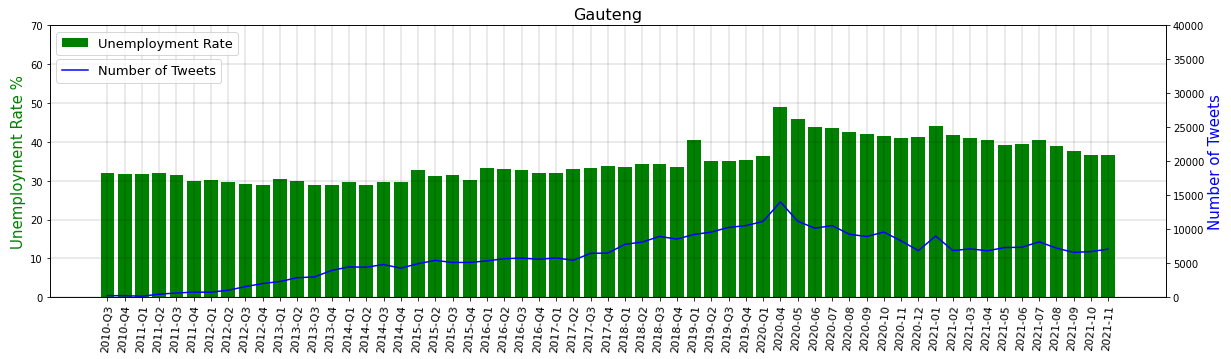


(D) Gauteng


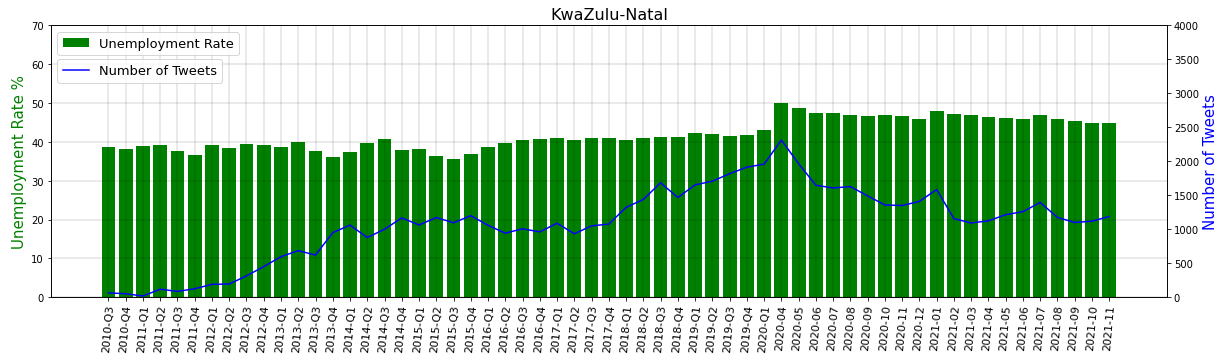


(E) KwaZulu-Natal


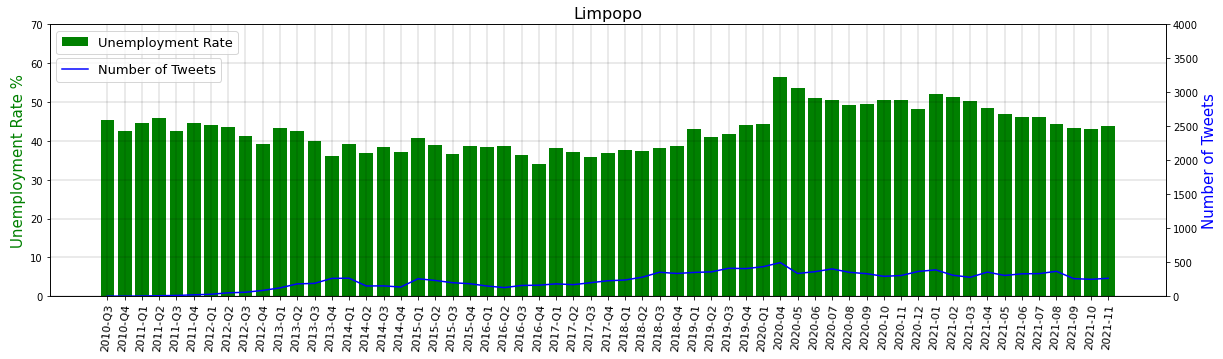


(F) Limpopo


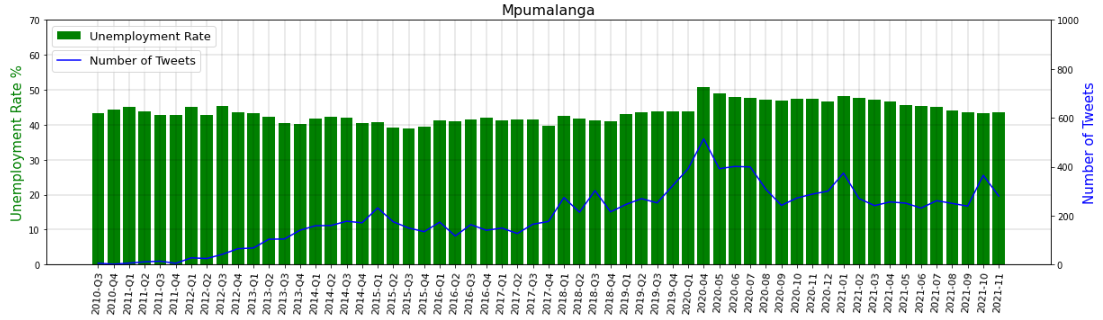


(G) Mpumalanga


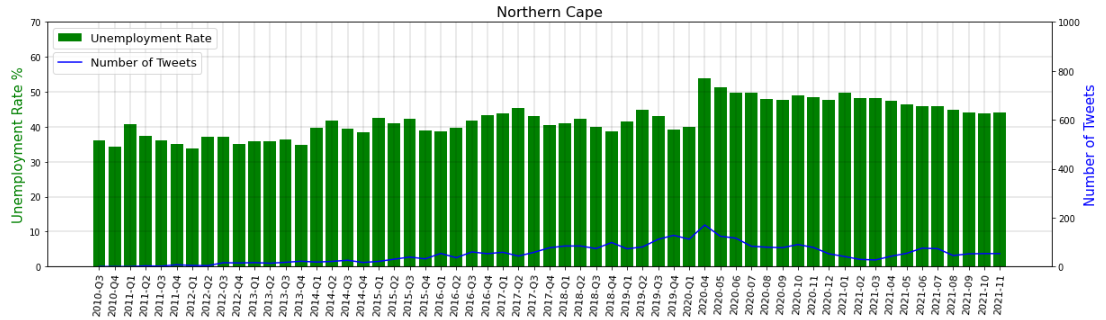


(H) Northern Cape


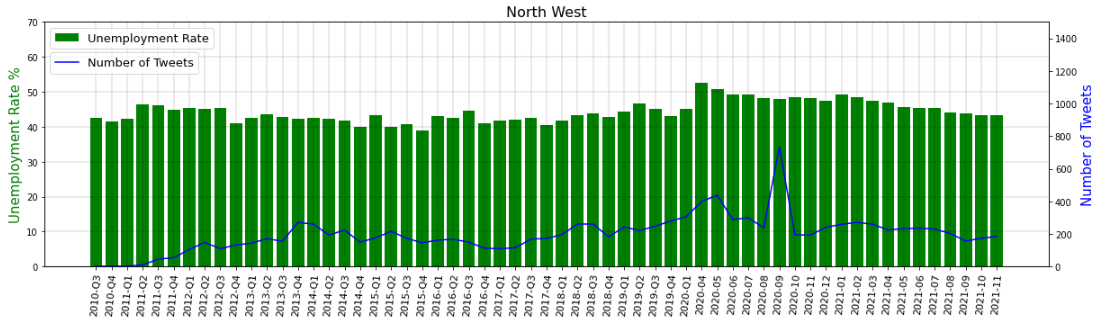


(I) North West


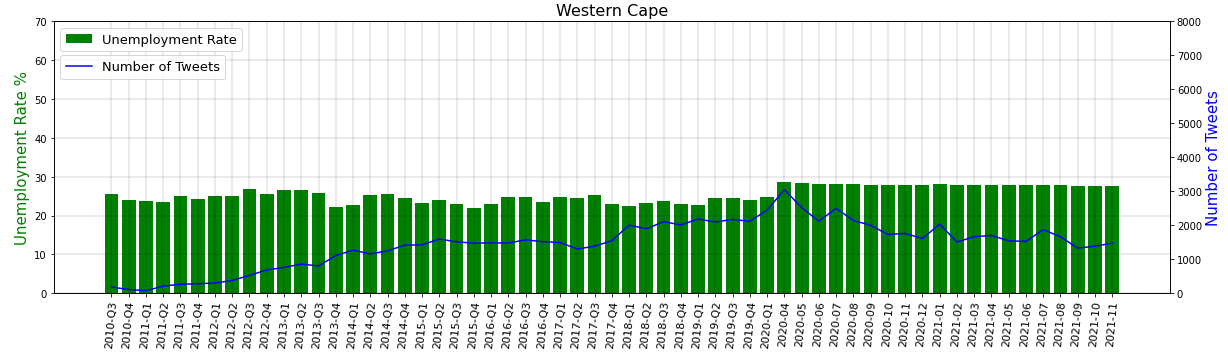


(J) Western Cape

**Figure A.2:** Comparing unemployment rate with the number of tweets for the whole country and different provinces

We use our PCR model to nowcast the unemployment rate of different provinces. Figure A.3 shows the correlation of the actual and estimated unemployment rates for different provinces with 95 percentiles.


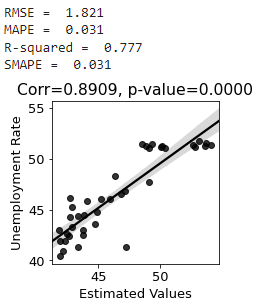

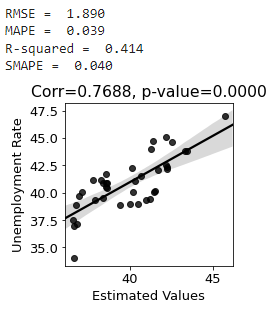

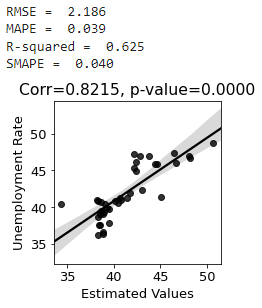


(A) Eastern Cape (B) Free State (C) KwaZulu-Natal


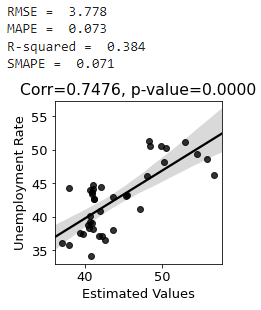

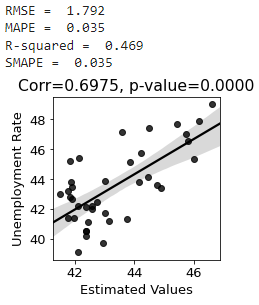

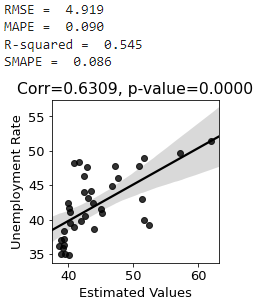


(D) Limpopo (E) Mpumalanga (F) Northern Cape


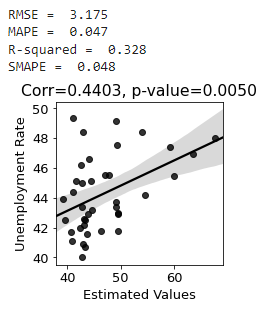

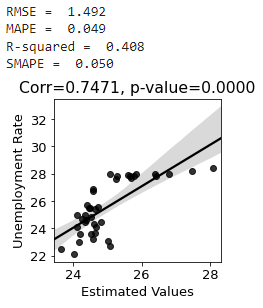


(G) North West (H) Western Cape

**Figure A.3:** Correlation between predicted and actual values of unemployment rate and their metrics for different provinces with 95 percentiles

Moreover, we use one third of the data before COVID-19 for KwaZulu-Natal to nowcast the unemployment rate of this province during COVID-19 pandemic. Figure A.4 shows that the predicted values closely follow the actual unemployment rate, and Figure A.4 shows that the predicted values are very well correlated with the actual values and have reasonable metrics.


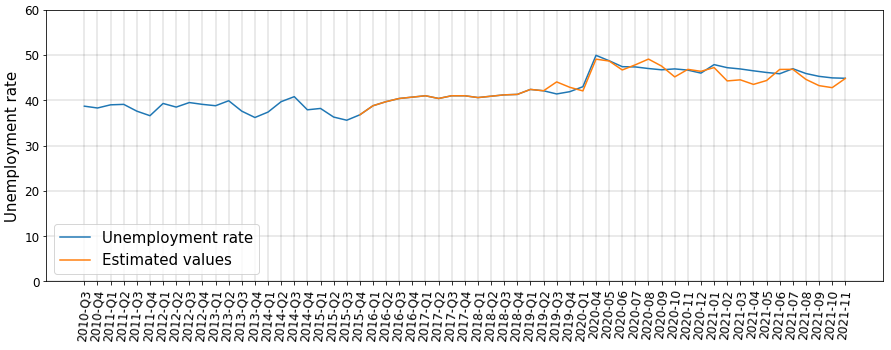

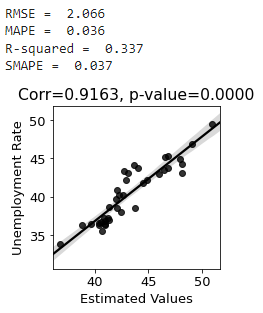


(A) (B)

**Figure A.4:** (A) The estimated values of unemployment rate in KwaZulu-Natal closely follow it actual values, (B) Correlation between the estimated values of the unemployment rate during COVID-19 and the actual unemployment rate for KwaZulu-Natal province with 95 percentiles

**Table A.1:** Keywords used for gathering the dataset

| Keyword | Corr. before COVID-19 | P-Value before COVID-19 | Corr. during COVID-19 | P-Value during COVID-19 | Select |
| --- | --- | --- | --- | --- | --- |
| lost * job (“lost his job”, “lost her job”, “lost my job”, and “lost their jobs”) | 0.69 | <.001 | 0.78 | <.001 | X |
| jobless | 0.47 | .002 | 0.53 | .01 | X |
| different forms of retrench (“retrench”, “retrenches”, “retrenched”, “retrenching”, “retrenchment”, and “retrenchments”) | 0.73 | <.001 | 0.72 | <.001 | X |
| different forms of layoff (“layoff”, “lay off”, “lay-off”, “laid off”, “laid-off”, “laid her off”, “laid him off”, “laid me off”, and “laid them off”) | 0.34 | .02 | 0.77 | <.001 |  |
| employed | 0.65 | <.001 | 0.72 | <.001 | X |
| unemployed | 0.64 | <.001 | 0.68 | <.001 | X |
| employment | 0.71 | <.001 | 0.16 | .48 |  |
| unemployment | 0.7 | <.001 | 0.07 | .75 |  |
| debt | 0.62 | <.001 | 0.81 | <.001 | X |
| bankrupt or bankruptcy | 0.52 | <.001 | 0.71 | <.001 | X |
| loan | 0.5 | .001 | 0.69 | <.001 | X |
| famine | 0.49 | .001 | 0.77 | <.001 | X |
| inflation | 0.39 | .01 | 0.43 | .05 |  |
| broke (“be broke”, “being broke”, “am broke”, “are broke”, and “is broke”) | 0.45 | .003 | 0.48 | .02 | X |
| career | 0.46 | .002 | 0.83 | <.001 | X |
| hire | 0.61 | <.001 | 0.59 | .006 | X |
| money | 0.54 | <.001 | 0.88 | <.001 | X |
| payment | 0.66 | <.001 | 0.85 | <.001 | X |
| afford | 0.57 | <.001 | 0.92 | <.001 | X |
| buy | 0.48 | .001 | 0.87 | <.001 | X |
| rent | 0.68 | <.001 | 0.83 | <.001 | X |
| lend | 0.46 | .002 | 0.83 | <.001 | X |
| poverty | 0.54 | <.001 | 0.51 | .02 | X |
| axed | 0.25 | .11 | 0.63 | .002 |  |
| canned | 0.51 | .002 | 0.72 | <.001 | X |
| downsized | 0.35 | .02 | 0.41 | .06 |  |
| outsourced | 0.41 | .008 | 0.27 | .23 |  |
| pink slip | -0.01 | .94 | 0.09 | .69 |  |
| “fired job” or “been fired” | 0.24 | .12 | 0.34 | .14 |  |

**Table A.2:** Correlation and its p-values between the number of tweets and unemployment rate for the whole country and different provinces

| **Location** | **Corr. with Unemployment Rate** | **P-Value** |
| --- | --- | --- |
| South Africa | 0.72 | <.001 |
| Eastern Cape | 0.72 | <.001 |
| Free State | 0.69 | <.001 |
| Gauteng | 0.81 | <.001 |
| KwaZulu-Natal | 0.67 | <.001 |
| Limpopo | 0.47 | <.001 |
| Mpumalanga | 0.51 | <.001 |
| Northern Cape | 0.62 | <.001 |
| North West | 0.48 | <.001 |
| Western Cape | 0.27 | .03 |
